# Impact of Health Education on Knowledge and Behaviors toward Infectious Diseases among Students in Gansu Province, China

**DOI:** 10.1155/2018/6397340

**Published:** 2018-03-07

**Authors:** Manli Wang, Xuemei Han, Haiqing Fang, Chang Xu, Xiaojun Lin, Shuxu Xia, Wenhan Yu, Jinlu He, Shuai Jiang, Hongbing Tao

**Affiliations:** ^1^School of Medicine and Health Management, Tongji Medical College, Huazhong University of Science and Technology, Wuhan, Hubei, China; ^2^School of Public Health, Lanzhou University, Lanzhou, Gansu, China; ^3^Administration Office, Shenzhen People's Hospital, Second Clinical Medical College of Jinan University, Shenzhen, Guangdong, China

## Abstract

**Objectives:**

Infectious disease knowledge and behaviors are key elements that ensure student health and safety. This study explores the impact of health education on student knowledge and behaviors toward infectious diseases and determines the factors affecting infectious diseases knowledge and behaviors among students in Gansu, China.

**Methods:**

A cross-sectional study and three sampling methods were used in two counties, 12 schools, and 32 classes in Gansu, China, from 2012 to 2013. Collected data included the following: (1) sociodemographic characteristics of 2002 students (1001 participants in the intervention group and 1001 in the control group); (2) accuracy of student knowledge and behaviors toward infectious diseases based on comparison of intervention and control groups through *X*^2^ test; and (3) mean scores on knowledge and behavior of students with different characteristics toward infectious diseases, as analyzed through analysis of variance (ANOVA). Multiple linear regression was conducted to analyze factors affecting student knowledge and behaviors toward infectious diseases.

**Results:**

Statistically significant differences were observed among eight items of infectious disease transmission and treatment knowledge between intervention and control groups (*P* < 0.001). Average accuracies of knowledge and behaviors toward infectious diseases reached 72.23% and 60.03%. Significant differences were observed in six items on student behavior in rural and urban areas (*P* < 0.001). Health education, household register, and county affected scores of student knowledge and behaviors toward infectious diseases (*P* < 0.05). Gender and education level also affected scores of student behaviors toward infectious diseases (*P* < 0.001).

**Conclusions:**

Health education contributes to student knowledge and behaviors toward infectious diseases. Students in the control group need intensified health education on infectious diseases. Health education needs to pay particular attention to rural students, all male students, and students at senior high school level living on campus.

## 1. Introduction

Internationally, infectious diseases include many types, among which tuberculosis (TB), influenza, and mumps are relatively common [1]. Although the worldwide incidence of TB slowly decreases, the global disease rate remains substantial with 9 million cases and 1.5 million deaths in 2013 [2]. China is one of the 22 countries in the world that feature a high TB rate, with incidence in 2015 reaching 63.4 out of 10,000 and mortality in 2014 totaling 2.32 out of 100,000 [3]. In China, influenza is highly infectious and strongly epidemic or pandemic among young people [4]. Mumps is a common respiratory infectious disease in children and adolescents [5]. In the United States, the incidence of mumps in 1986 and 1987 increased threefold compared with the average incidence from five years earlier. In China, the incidence of mumps continually and steadily rose over the past decade [6]. These three major infectious diseases cause serious impact and harm, leading to adoption of measures that control spread of imminent infectious diseases.

Primary, junior, and senior high school students form a special group that features high personnel density and close interpersonal contact, easily causing outbreaks of infectious diseases in the absence of timely control [7]. As a gathering place for young people, schools display characteristics of a largely susceptible population, frequent contact, and gathering age and are a place where outbreaks of infectious diseases, especially respiratory diseases, may occur. Coupled with rapid socioeconomic development and frequent population flows, infectious diseases more possibly occur and spread in schools. Epidemics or outbreaks of infectious diseases in schools not only affect teaching order, resulting in an adverse social effect, but also negatively affect physical and mental health of young people [8, 9]. Studies showed that more than 70% of public health emergencies in China occur in schools, with most emergencies being infectious disease events [10]. Therefore, strengthening the attention of students and schools presents significance in preventing and treating infectious diseases.

Health literacy is often indicated to accommodate an individual approach by substituting the three domains of health “healthcare, disease prevention, and health promotion” with “being ill, being at risk, and staying healthy” [11]. Health literacy bears significance in improving prevention and control of infectious diseases, whereas health knowledge and behavior are important components of health literacy. Given the current high incidence of infectious diseases among primary, junior, and senior high school students, improving health literacy of students on infectious diseases serves as an important channel in controlling epidemics and outbreaks of infectious diseases in schools. Health education can improve student knowledge on infectious diseases and promote the development of appropriate behaviors toward infectious disease prevention and control. Health promotion is based on health education, which is founded on health knowledge. Health education effectively slows spread of infectious diseases, and conducting school health education programs not only provides students with proper knowledge and behavior toward infectious diseases but also benefits the comprehensive development of schools [12]. Therefore, health education must be strengthened to improve health literacy of students.

Numerous scholars studied the effects of health education in influenza, TB, and mumps on improvement of knowledge on infectious diseases and changes in preventive behaviors among school students [13]. Li et al. studied influenza A (H1N1) awareness among medical college students before and after a health education program and observed that health education is the main approach for medical college students to accept scientific and specific knowledge on influenza A (H1N1) prevention. Mohammadi et al. [14], Wilches et al. [15], and Juniarti et al. [13] explained and affirmed effects of educational program on knowledge attitudes and preventive behaviors toward TB among students and adults. In 2013, Luo et al. [16] studied changes in awareness in measles, rubella, and mumps among middle school students in Tianjin before and after health education intervention and confirmed that health education can improve cognitive level and prevention awareness of infectious diseases, such as mumps. In 2015, Yue [17] discussed impacts of face-to-face health education on knowledge of primary school students on mumps and observed that health education can significantly improve mumps awareness of primary school students. Numerous empirical studies also showed that health education can change unhealthy attitudes and behaviors, effectively curbing infectious diseases and epidemics [14, 18]. Previous studies mainly stated the positive effect of health education on prevention and control of infectious diseases among students but rarely explored correct levels of knowledge and behavior of students with different social backgrounds toward infectious diseases; only a small number of studies reported factors that affect student knowledge and behavior toward infectious diseases.

Gansu is a province located in a remote area of Western China and features relatively backward level of socioeconomic development and inadequate education and health resources [19]. This paper aims to evaluate the effects of a comprehensive health education intervention project on infectious diseases in Gansu, China. Based on the understanding of status quo of knowledge and prevention of influenza, TB, and mumps among primary, junior, and senior high school students in two counties of Gansu, we compared accuracy and scores of students before and after a health education intervention and explored factors behind student knowledge and behavioral scores to provide empirical reference for infectious disease prevention among pupils.

## 2. Methods

### 2.1. Design

This research is a cross-sectional study designed to evaluate the effects of health education on knowledge and behaviors of primary, junior, and senior high school students toward infectious diseases.

### 2.2. Settings and Samples

**Figure 1:**
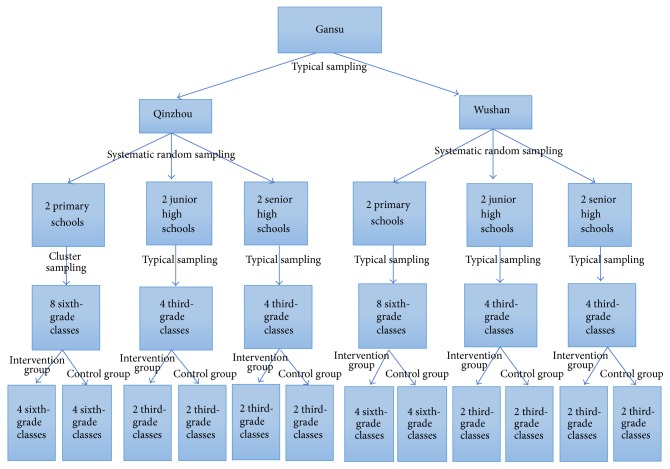
Settings and study samples.

The target population of this study comprised primary, junior, and senior high school students enrolled in various school classes in Qinzhou and Wushan counties in Gansu, China. Qinzhou county is located in the southeastern part of Gansu province, with a total area of 2442 Km^2^. In 2012, Qinzhou county had a total population of 690,000, its region's social productive capital was 1.82 billion dollars (Ren Min Bi), and the urban residents' per capita disposable income reached 2048.49 dollars; the per capita cash income of farmers reached 557.31 dollars. Wushan county is located in southeastern part of Gansu Province. In 2012, the total area of Wushan county was 2011 Km^2^, and the total population was 475,500. The county's social productive forces were 0.59 billion dollars, the urban residents' per capita disposable income reached 2016.23 dollars, and the per capita net income of farmers was 580.80 dollars. Three sampling methods were used to select samples.  Figure 1 indicates detailed settings and the sampling process.

Before the implementation of the infectious disease health education intervention, we first identified the intervention group and the control group. In the identified classes, we allocated the students in the intervention and control groups with the random cluster sampling method. In Qinzhou county, for the 8 sixth-grade primary school student classes, 4 classes were randomly allocated to the intervention group and the other 4 classes were regarded as the control group students; for the 4 third-grade junior high school student classes, 2 classes were randomly allocated to the intervention group, and the other 2 classes were regarded as the control group students; for the 4 third-grade senior high school student classes, 2 classes were randomly allocated to the intervention group and the other 2 classes were regarded as the control group students. Similar to the students in the Qinzhou county, we also allocated the students to the intervention group and the control group randomly. Hence, there were 8 sixth-grade primary school student classes, 4 third-grade junior high school student classes, and 4 third-grade senior high school student classes. The distribution of students and classes in the control group was the same as in the intervention group (Figure 1).

### 2.3. Instrument

A questionnaire was self-designed based on the Center for Health Education of China to assess knowledge and behaviors of sampled students toward infectious diseases. The questionnaire consisted of three parts: (1) selected student demographic information in sample areas, (2) nine-item questions related to infectious disease knowledge, and (3) six-item questions concerning behaviors for preventing infectious diseases. Various options were used to assess students' responses to each question.

A pilot study was conducted on primary, junior, and senior high school students (*n* = 100) who were not included in samples; modifications of the instrument and method were accordingly performed. Internal consistency reliability (Cronbach's alpha) estimates reached 0.90 for infectious disease knowledge and 0.95 for infectious disease prevention behaviors. The overall internal consistency reliability totaled 0.93.

### 2.4. Data Collection Method

After obtaining official permission from various schools, we carried out a monthly health education program on infectious diseases in classes of intervention groups from 2012 to 2013. We held the program 12 times, with topics focusing on playing promotional cartoons of infectious disease awareness, implementing lectures by professional medical staff, releasing handbook copies on mumps, TB, and influenza, and organizing blackboard newspapers, hand copy newspapers, and publicity columns. Students in the control group did not receive any intervention measures.

Before the health education intervention, we did not collect any data related to the infectious diseases knowledge and behavior of the sample students. Six months after the implementation of the health education intervention project on infectious diseases [20, 21], trained and qualified investigators were sent to each sample area to administer questionnaires. Questionnaires were distributed to students who were selected to participate in the study and were readministered to students in both groups during a meeting that was held two months after the end of the health education program. The intervention group included 1001 students (433, 269, and 299 in primary, junior, and senior high school levels, resp.), whereas the control group comprised 1001 participants (384, 301, and 316 in primary, junior, and senior high school, resp.). Both control and intervention groups yielded a response rate of 100%.

### 2.5. Quality Control of the Questionnaires Survey

In the process of our research, in order to ensure the reliability and truthfulness of the survey results, we did the following work. (1) In the questionnaire design, we did a good job preplanning, standardized language, and cleared the type of subjects; we arranged the order of the problems and carried out a preinvestigation and assessment. (2) In order to ensure the investigator's ability to perform, after training the investigators, we strictly supervised and assessed the work of the investigators in the course of the investigation. (3) After selecting the survey objects, in order to ensure the quality of the questionnaires, we gave the objects a certain kind of material reward (such as a pen) and gave them plenty of time to answer the questions. (4) We tested the reliability of the questionnaires to ensure quality. (5) After calling in all the questionnaires, we checked and removed the unqualified questionnaires in time. (6) We adopted the data double-entry method to carry out logical errors correction to ensure the quality of data entry.

### 2.6. Data Analysis

Data entry was performed using Microsoft Excel 2013 (Microsoft Office, Redmond, WA, USA) and EpiData 3.1 (EpiData Association, Odense, Denmark). Double-entry method was adopted to ensure accuracy, and SPSS 19.0 statistical software (IBM Company, Armonk, NY, USA) was used for statistical analysis. Sociodemographic characteristics of sample students were defined through descriptive statistics. *X*^2^ test was applied to analyze and compare accuracy of knowledge and preventive behavior toward infectious diseases between intervention and control groups. ANOVA was adopted to compare mean scores on knowledge and prevention behaviors of intervention and control groups under different sociodemographic conditions in sample areas. Multiple linear regression was used to analyze factors that influence infectious disease knowledge and behaviors of sample students.

### 2.7. Ethics Approval

This research was approved by the Research Ethics Committee of the School of Medicine and Health Management, Tongji Medical College, Huazhong University of Science and Technology. All participants indicated their willingness to participate in this research.

## 3. Results

### 3.1. Sociodemographic Characteristics of Sample Students

Table 1 describes sociodemographic characteristics of samples and shows similar characteristics of both intervention and control groups compared with those of the total number of participants. Most of the comparisons between the intervention and control groups have no statistical significance.

### 3.2. Accuracy of Knowledge and Behavior of Sample Students toward Infectious Diseases

Table 2 shows accuracy of knowledge and behaviors of sample students toward infectious diseases in the intervention and control groups.

In terms of knowledge on infectious diseases, accuracies of all nine items in the intervention group were higher than those in the control group. Except for item (6), which asks “Whether the examination and treatment of TB is free in China,” results of other items statistically differed between these two groups (*P* < 0.001). The intervention group featured higher average accuracy than the control group. The three items with the lowest accuracies were items (3), (6), and (9) for the total sample, items (6), (8), and (9) in the intervention group, and items (1), (3), and (9) in the control group.

With regard to the preventive behavior toward infectious diseases, accuracies of all six items in the intervention group were higher than those in the control group. On average, these two groups presented a statistically significant difference (*P* < 0.001). The intervention group yielded a higher average accuracy than the control group. Among the six mentioned items, items with the lowest accuracies included items (1), (4), and (6) for the total sample, items (1), (4), and (6) in the intervention group, and items (1), (5), and (6) in the control group.

### 3.3. Scores on Knowledge and Behavior of Sample Students toward Infectious Diseases

Different knowledge and preventive behavior scores among sample students were analyzed using ANOVA.

In terms of knowledge scores on infectious diseases, the intervention group obtained a higher score than the control group, and the difference was statistically significant (*P* < 0.001). Knowledge scores of Han students were statistically lower than those of minority students (*P* < 0.05). Urban students showed higher scores than rural students (*P* < 0.001); students from Qinzhou yielded higher scores than those from Wushan county (*P* < 0.001). Statistically significant differences were noted in knowledge scores among primary, junior, and senior high school students with respect to gender, age, and educational levels.

In terms of behavior scores on infectious diseases, scores of the intervention group were higher than those of the control group, and the difference was statistically significant (*P* < 0.001). Scores of male students were statistically lower than those of female students (*P* < 0.001). Students with different ages exhibited statistically different prevention behavior scores (*P* < 0.001), with 14–17- and 18–20-year-old students scoring lower than 10–13-year-old students (*P* < 0.01, *P* < 0.001). Scores among students from different age groups were statistically significant (*P* < 0.001). Primary and junior high school students obtained higher scores than senior high school students (*P* < 0.001, *P* < 0.001). Urban students yielded higher scores than rural students (*P* < 0.001). Students from Qinzhou featured higher scores than those from Wushan county (*P* < 0.001).

### 3.4. Factors of Infectious Disease Knowledge and Behaviors among Sample Students

We applied the multiple linear regression method in Table 4 to determine factors affecting knowledge and preventive behaviors of participants toward infectious diseases.

In terms of factors that affect student knowledge on infectious diseases, a significant difference existed between control and intervention groups, with the latter serving as a reference sample area (*P* < 0.001). In terms of educational level, using the former as a reference, a statistically significant difference was observed between primary and senior high school groups (*P* < 0.001). In terms of household register, statistical significance was observed between urban and rural groups, with the former as a reference (*P* < 0.001). In terms of county, a significant difference existed between Qinzhou and Wushan groups (*P* < 0.001), with the former serving as a reference.

In terms of factors that affect preventive behaviors toward infectious diseases, the control group significantly differed from the intervention group; the latter served as a reference sample (*P* < 0.001). In terms of gender, a significant difference was observed between male and female groups, with the former acting as a reference (*P* < 0.001). In terms of ethnicity, statistical significance was present between the Han group and the minority group based on the former as a reference (*P* < 0.05). In terms of household register, the urban group, which served as a reference, statistically differed from the rural group (*P* < 0.001). In terms of county, a significant difference existed between Qinzhou and Wushan groups (*P* < 0.001), with the former serving as a reference.

## 4. Discussion

This study compared accuracy and scores in knowledge and behavior toward infectious diseases of control and intervention student groups from Gansu, China. Our study discovered that both accuracy and scores of the intervention group were higher than those of the control group, and the statistical difference between the groups was significant (*P* < 0.001). Therefore, the intervention program that promoted health education of major infectious diseases effectively spread knowledge and information on behavior toward infectious diseases among primary, junior, and high school students. These results were similar to research studies of Kang et al. [22], Al-Mazrou et al. [23], and Saleh et al. [24].

Our study also showed that the intervention program, namely, health education, was a positive factor that affected scores of infectious disease knowledge and prevention behavior among primary, junior, and high school students in Gansu, China, further affirming the value of health education. On the one hand, students in schools are mainly engaged in learning basic knowledge and applying this knowledge to examinations [25]; these students lack the initiative and enthusiasm for learning specialized infectious disease courses. On the other hand, a majority of primary, junior, and senior high school students did not take a detailed course on infectious diseases, resulting in their lack of knowledge in this field. Relevant studies [13, 26] showed that implementation of health education for school students was conducive for students to consciously adopt healthy behaviors and lifestyle, eliminating or mitigating risk factors that affect spread of infectious diseases, preventing infectious diseases, and promoting health and improving quality of life. Therefore, we believe in the significance of conducting health education on infectious diseases among primary, junior, and high school students to further improve their knowledge on prevention of infectious diseases and their overall health quality.

In terms of knowledge and behavior on major infectious diseases, both intervention and control groups scored the lowest on item (9) of infectious disease knowledge and items (1) and (6) of infectious disease behavior. These items comprised topics that students mainly lacked awareness of. Results were similar to those of research by Tuohetamu et al. on influenza awareness of students [4]. Similarly, relatively low accuracies of knowledge and behavior items toward infectious diseases were noted in the intervention group and control group. These results suggest that, with regard to the low accuracy items, primary, junior, and senior high school students possess insufficient knowledge of preventive knowledge and behaviors on infectious diseases. This observation should also concern researchers and educators. Similarly, guidance and education on health behavior should be given to students to improve their knowledge and behavior in preventing infectious diseases.

Comparison of student scores on knowledge of infectious diseases showed statistical significance in the aspect of different home addresses of students (*P* < 0.001); urban students scored higher than rural students. Therefore, home address is an important factor that influenced knowledge of student participants. In general, Chinese urban households attained higher economic, domestic, and parental education levels than rural households [27], and the former featured more educational advantages than the latter. High levels of economic and educational status translate to high levels of health knowledge [28]. In the more favorable urban families, students showed more opportunities of receiving knowledge on infectious diseases, resulting in their relatively high knowledge scores on infectious diseases. This observation is similar to research results of Chen et al. [29], who analyzed knowledge, behavior, and intervention needs of primary school students on infectious diseases.

Knowledge scores between the two different counties were also statistically different (*P* < 0.001). In the sample area, scores of primary, junior, and senior high school students in Qinzhou were higher than those in Wushan; and results are similar to those of previous research by Hu et al. on health literacy of Chinese high school students [30]. Therefore, educators should emphatically pay attention to education of counties with low levels of knowledge on infectious diseases. Simultaneously, higher-scoring areas should share their health education experiences with schools in lower-scoring areas to promote common improvement of knowledge on infectious disease within provincial or national areas.

As shown in Tables 3 and 4, comparisons of infectious disease prevention behavior scores between students of different genders were statistically significant (*P* < 0.001), with female students scoring higher than male students. Hence, gender is also an important factor affecting infectious disease prevention behavior of students. One possible reason is attributable to the difference between general characters of females and males, with females being more delicate than males and paying more attention to personal health care. Therefore, female students show a higher level of health awareness [31] and more correct infectious disease prevention behavior. Taylor [32] also noted that females feature higher awareness of TB than males based on the study of gender differences in health literacy of the disease. Therefore, we advocate implementation of peer education [33] among students to improve the overall level of infectious disease prevention behavior of students.

Our results also indicated statistically different infectious disease behaviors among primary, junior, and senior high school students with different ages and education levels (*P* < 0.001). Younger students who have a lower educational level obtained higher scores. A majority of young students in China live with their parents and easily accept the influence of their families on infectious disease prevention, resulting in their relatively high behavior scores on infectious diseases. Upon elevation of education level, most Chinese students live as boarders where learning pressure increases [34]. Given the lack of family health guidance and heaviness of learning task at this stage, students with higher grades pay less attention to infectious disease prevention. Therefore, students who incur higher grades and education levels and are older obtained lower behavior scores on infectious diseases. Therefore, researchers and educators should not only pay attention to health education of primary school students but also emphasize on health education of junior and senior high school students.

Similar to infectious diseases knowledge scores, differences in infectious disease prevention behavior scores of students from different family addresses were statistically significant (*P* < 0.001), where urban students scored higher than rural students. At the same time, students from different county areas also yielded statistically different scores on infectious disease prevention behavior (*P* < 0.001); Qinzhou county students obtained higher behavior scores than Wushan county similar to results on knowledge scores. Family address and county area are significant factors that influence scores of infectious disease prevention behavior among primary, junior, and senior high school students, and this finding should draw the attention of educators and decision-makers.

## 5. Limitation

Our study features some limitations. First, this article only selected two counties in Gansu province as research sites, resulting in the external validity of this study being not good. Therefore, promotion and popularization of research results should consider geographical restrictions. Second, we only collected the data of the intervention group and the control group after the intervention, but we did not collect the data before the intervention. This study compared infectious disease knowledge and prevention behaviors in terms of accuracy and scores in health education of the intervention and control groups but did not study knowledge- and prevention-related behavioral changes of student groups before and after health education intervention. The lack of infectious diseases knowledge and behavior assessment of the two groups before the health education project may lead to a lack of more effective evidence of health education's effect in this study. Hence, difference-in-differences model should be used in future studies to empirically verify effects of health education on infectious diseases on students. Third, this research lacked the comparison between the results of our self-designed questionnaires and the HLS-EU instrument, resulting in a certain lack of richness and perfection. For future research, a pre/post study can be performed. In addition, a study using the HLS-EU instrument alongside the current instruments could explore if there is an association between “general” health literacy levels and knowledge of infectious diseases. Fourth, due to many factors, the survey results easily deviate from the real situation. Although we controlled the quality of the questionnaires by means of strict questionnaire design, improved the investigator's ability to perform, improved the coordination degree of the survey objects, and tested the reliability of the questionnaires, the questionnaire results may still have a certain deviation from the real situation. However, this deviation does not affect the overall content and conclusions of the findings on the whole. Fifth, the duration of this study is 6 months, so there may be some limitations with the time frame. Shorter or longer periods of time are likely to affect the impact of health education programs on students' knowledge and behavior toward infectious diseases. However, this study has referred to the extant literature to determine the time period of 6 months [20, 21], so there is a rationale for the time frame chosen. Despite these limitations, this study still proves its significance in health education on infectious diseases and provides reference for promoting knowledge and preventive behaviors among primary, junior, and senior high school students.

## 6. Conclusion

In terms of knowledge and preventive behaviors toward infectious diseases, primary, junior, and senior high school students in health education intervention group showed higher accuracy and scores than those in the control group. The knowledge and behavior for preventing infectious diseases of students in the control group should be strengthened through health education toward infectious diseases. Simultaneously, more focus should be given to students who belong to ethnic minorities, living in rural areas, and males. Students with high grades and educational levels scored lower in knowledge and behavior toward infectious diseases. Hence, more attention to health education of infectious diseases should be paid to students who receive high grades and educational level. The government, society, medical institutions, and schools should also collaborate in promoting infectious disease awareness and behavior of students [35, 36].

Our study takes students as a research sample, covering all kinds of pupils of primary, junior, and senior high school students, which is different from the previous research in the same field. Additionally, this study not only compared the differences of accuracy rate of infectious diseases knowledge and behavior between the intervention and control groups, but also compared their infectious disease knowledge and behavior scores and explored the factors impacting these scores. Therefore, this research's content is much richer and has some innovation. Consequently, our research has some innovations in the study samples and study contents. At the practical level, this study may provide some guidance for the design and carrying out of the health education project toward infectious diseases for the students. At the theoretical level, our study may also provide some reference on design, sample choosing, and methods application for the research related to effect evaluation of health education toward other important infectious diseases on students or other people in a certain degree. In addition, this study is helpful in that it provides reference for the health education of infectious diseases in other cities and regions of China and in developing countries. Moreover, the limitations of this study will provide some research ideas and direction for future studies.

## Figures and Tables

**Table 1:** Sociodemographic characteristics of the investigated sample students.

Items	Kinds	Total		Intervention group		Control group		*X* ^*2*^	*P*
		*N* = 2002	%	*n*1 = 1001	%	*n*2 = 1001	%		
Gender	Male	1076	53.75	541	54.05	535	53.45	0.072	0.823
	Female	926	46.25	460	45.95	466	46.55		

Age	10–13 years old	798	39.86	424	42.36	374	37.36	8.312	0.016
	14–17 years old	906	45.25	421	42.06	485	48.45		
	18–20 years old	298	14.89	156	15.58	142	14.19		

Nationality	Han	1026	51.25	543	54.25	483	48.25	5.611	0.028
	Minority	976	48.75	458	45.75	518	51.75		

Education level	Primary school	817	40.81	433	43.26	384	38.36	5.205	0.074
	Junior high school	570	28.47	269	26.87	301	30.07		
	Senior high school	615	30.72	299	29.87	316	31.57		

Household register	Urban	1407	70.28	756	75.52	651	65.03	424.153	*P* < 0.001
	Rural	595	29.72	245	24.48	350	34.97		

County	Qinzhou	1002	50.05	501	50.05	501	50.05	<0.001	1.000
	Wushan	1000	49.95	500	49.95	500	49.95		

**Table 2:** Accuracy rate on infectious disease knowledge and behavior of sample students (*n*/%).

Items	Total (*N* = 2002)	Intervention group (*n*1 = 1001)	Control group (*n*2 = 1001)	*X* ^2^	*P*
*Accuracy rate of infectious disease knowledge*					
(1) Which of the following are infectious diseases?	1306 (65.23)	968 (96.70)	338 (33.77)	874.16	*P* < 0.001^*∗∗∗*^
(2) What's the transmission way of the mumps?	1449 (72.38)	988 (98.70)	461 (46.05)	695.72	*P* < 0.001^*∗∗∗*^
(3) What methods can prevent mumps?	1263 (63.09)	974 (97.30)	289 (28.87)	1006.46	*P* < 0.001^*∗∗∗*^
(4) What's the transmission way of the TB?	1800 (89.91)	995 (99.40)	805 (80.42)	197.77	*P* < 0.001^*∗∗∗*^
(5) What are the symptoms of TB?	1889 (94.36)	1000 (99.90)	889 (88.81)	115.56	*P* < 0.001^*∗∗∗*^
(6) Whether the examination and treatment of TB is free in China?	1190 (59.44)	612 (61.14)	578 (57.74)	2.40	0.122
(7) Can the TB be cured?	1711 (85.46)	961 (96.00)	750 (74.93)	180.07	*P* < 0.001^*∗∗∗*^
(8) What's the transmission way of the flu?	1468 (73.33)	946 (94.51)	522 (52.15)	460.39	*P* < 0.001^*∗∗∗*^
(9) What methods can prevent flu?	936 (46.75)	817 (81.62)	119 (11.89)	977.56	*P* < 0.001^*∗∗∗*^

Average accuracy	72.23	91.67	52.74		

*Accuracy rate of infectious disease behaviors*					
(1) Do you wash your hands before having a meal?	1064 (53.15)	675 (67.43)	389 (38.86)	164.97	*P* < 0.001^*∗∗∗*^
(2) Do you wash your hands after you go to the toilet?	1208 (60.34)	719 (71.83)	489 (48.85)	110.42	*P* < 0.001^*∗∗∗*^
(3) If you have phlegm, how do you usually deal with it?	1880 (93.91)	988 (98.70)	892 (89.11)	81.70	*P* < 0.001^*∗∗∗*^
(4) Whether you cover your nose and mouth when coughing or sneezing?	1192 (59.54)	714 (71.33)	478 (47.75)	115.49	*P* < 0.001^*∗∗∗*^
(5) If you found you have a fever in school, what should you do?	1253 (62.59)	885 (88.41)	368 (36.76)	570.18	*P* < 0.001^*∗∗∗*^
(6) If you suspect yourself having TB, where will you go to see a doctor?	614 (30.67)	492 (49.15)	122 (12.19)	321.13	*P* < 0.001^*∗∗∗*^

Average accuracy	60.03	74.48	45.59		

*Notes*. ^*∗∗∗*^*P* < 0.001.

**Table 3:** Scores of sample students in different items on infectious disease knowledge and behavior.

Items	Kinds	*n*	Scores on infectious disease knowledge				Scores on infectious disease behaviors				LSD
			Mean	SD	*F*	*P*	Mean	SD	*F*	*P*	
Group	Intervention group	1001	8.25	0.870	58.691	*P* < 0.001^*∗∗∗*^	4.47	1.308	28.367	*P* < 0.001^*∗∗∗*^	
	Control group	1001	4.75	1.675			2.74	1.418			

Gender	Male	1076	6.46	2.209	−1.016	0.310	3.46	1.637	−4.332	*P* < 0.001^*∗∗∗*^	
	Female	926	3.46	1.637			3.77	1.575			

Age	10–13 years old	798	6.55	2.238	0.331	0.719	3.78^a^	1.541	9.408	*P* < 0.001^*∗∗∗*^	a > b^*∗∗*^
	14–17 years old	906	6.46	2.151			3.54^b^	1.646			a > c^*∗∗∗*^
	18–20 years old	298	6.51	2.273			3.34^c^	1.673			b > c

Nationality	Han	1026	6.49	2.201	−2.327	0.020^*∗*^	3.60	1.612	−0.889	0.374	
	Minority	976	7.50	2.214			3.88	1.925			

Education level	Primary school	817	6.52	2.242	0.695	0.499	3.76^a^	1.543	16.156	*P* < 0.001^*∗∗∗*^	a > b
	Junior high school	570	6.56	2.068			3.71^b^	1.618			a > c^*∗∗∗*^
	Senior high school	615	6.42	2.274			3.30^c^	1.668			b > c^*∗∗∗*^

Household register	Urban	1652	6.90	2.122	18.850	*P* < 0.001^*∗∗∗*^	3.79	1.605	11.725	*P* < 0.001^*∗∗∗*^	
	Rural	350	4.63	1.527			2.71	1.347			

County	Qinzhou	1002	6.82	2.327	6.554	*P* < 0.001^*∗∗∗*^	4.02	1.605	11.892	*P* < 0.001^*∗∗∗*^	
	Wushan	1000	6.18	2.024			3.19	1.517			

*Notes.* ^*∗*^ *P* < 0.05; ^*∗∗*^*P* < 0.01; ^*∗∗∗*^*P* < 0.001.

**Table 4:** Multiple linear regression analysis of factors of infectious disease knowledge and behaviors among sample students.

Item	Infectious disease knowledge							Infectious disease behaviors						
	Unstandardized coefficients		Standardized coefficients	*T*	*P*	95% confidence		Unstandardized coefficients		Standardized coefficients	*T*	*P*	95% confidence	
	B	SE	Beta			Lower	Upper	B	SE	Beta			Lower	Upper
Constant	8.530	0.068		125.678	*P* < 0.001^*∗∗∗*^	8.397	8.663	4.950	0.067		74.197	*P* < 0.001^*∗∗∗*^	4.819	5.081
*Group*														
Intervention group	0^a^							0^a^						
Control group	−3.696	0.070	−0.839	−53.099	*P* < 0.001^*∗∗∗*^	−3.832	−3.559	−1.974	0.069	−0.611	−28.781	*P* < 0.001^*∗∗∗*^	−2.108	−1.839
*Gender*														
Male	0^a^													
Female	0.054	0.058	0.012	0.930	0.353	−0.060	0.168	0.256	0.057	0.079	4.463	*P* < 0.001^*∗∗∗*^	0.143	0.368
*Age*														
10–13 years old	0^a^							0^a^						
14–17 years old	−0.330	0.263	−0.053	−1.251	0.211	−0.846	0.187	−0.148	0.260	−0.033	−0.569	0.570	−0.657	0.361
18–20 years old	−0.251	0.243	−0.057	−1.033	0.302	−0.727	0.225	−0.106	0.239	−0.033	−0.444	0.657	−0.575	0.363
*Nationality*														
Han	0^a^							0^a^						
Minority	−0.116	0.257	−0.006	−0.450	0.652	−0.619	0.388	−0.607	0.253	−0.043	−2.403	0.016^*∗*^	−1.103	−0.112
*Education level*														
Primary school	0^a^							0^a^						
Junior high school	0.451	0.252	0.094	1.787	0.074	−0.044	0.945	−0.109	0.248	−0.031	−0.441	0.660	−0.596	0.378
Senior high school	0.503	0.244	0.103	2.063	0.039^*∗*^	0.025	0.982	0.185	0.240	0.052	0.768	0.442	−0.287	0.656
*Household register*														
Urban	0^a^							0^a^						
Rural	0.497	0.109	0.086	4.546	*P* < 0.001^*∗∗∗*^	0.283	0.712	0.664	0.108	0.156	6.170	*P* < 0.001^*∗∗∗*^	0.453	0.875
*County*														
Qinzhou	0^a^							0^a^						
Wushan	−0.821	0.070	−0.186	−11.800	*P* < 0.001^*∗∗∗*^	−0.957	−0.684	−1.069	0.069	−0.331	−15.599	*P* < 0.001^*∗∗∗*^	−1.203	−0.935

*Notes.* ^*∗*^ *P* < 0.05; ^*∗∗∗*^*P* < 0.001. ^a^This parameter is set to 0 because it is redundant.

## Abbreviations

TB: Tuberculosis
ANOVA: Analysis of variance.
